# Supplementary material for: DMRforPairs: identifying Differentially Methylated Regions between unique samples using array based methylation profiles
Source: BMC Bioinformatics. 2014 May 15;15:141. doi: 10.1186/1471-2105-15-141 (PMC4046028; doi:10.1186/1471-2105-15-141)
Supplement: Additional file 2 — DMRforPairs output for the comparison of A431-MCF7 and NA17018-NA17105. Please start from the HTML files in each folder. Available via the BMC Bioinformatics website. [file 1471-2105-15-141-S2.zip › 1394847754114233_MOESM2_ESM/A431_MCF7/figures/16380.pdf]

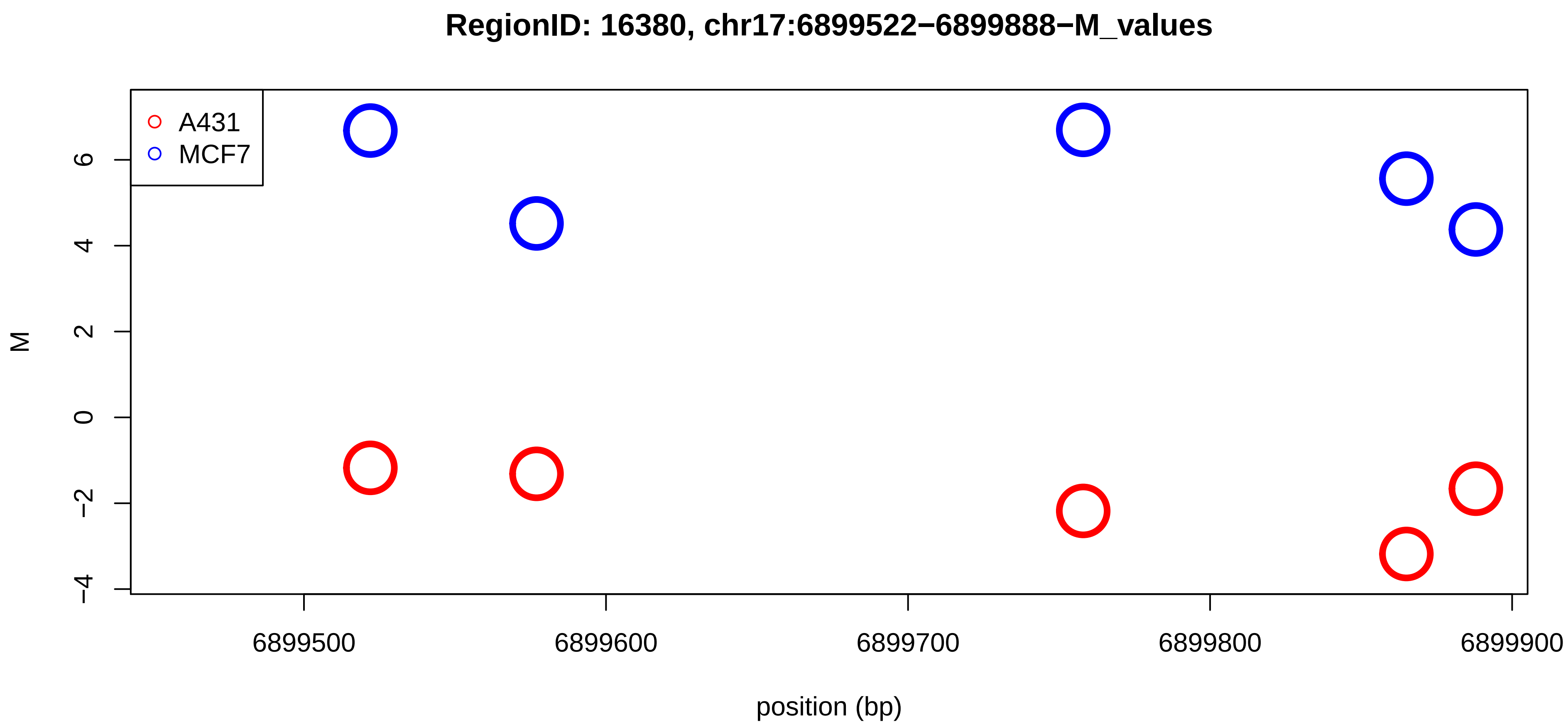
RegionID: 16380, chr17:6899522−6899888−M_values
A431
MCF7
M
6
4
2
0
−2
−4
6899500
6899600
6899700
6899800
6899900
position (bp)

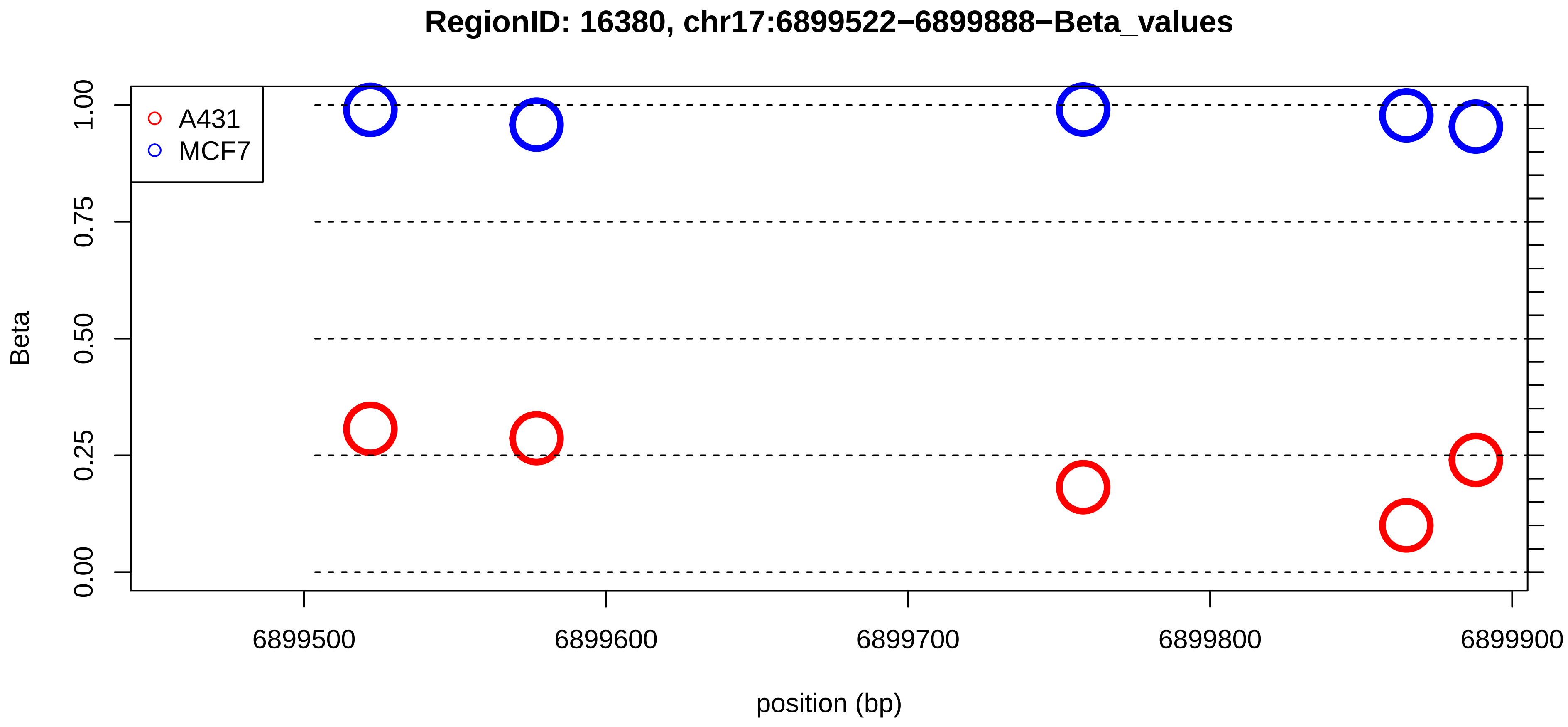
RegionID: 16380, chr17:6899522−6899888−Beta_values
A431
MCF7
Beta
1.00
0.75
0.50
0.25
0.00
6899500
6899600
6899700
6899800
6899900
position (bp)

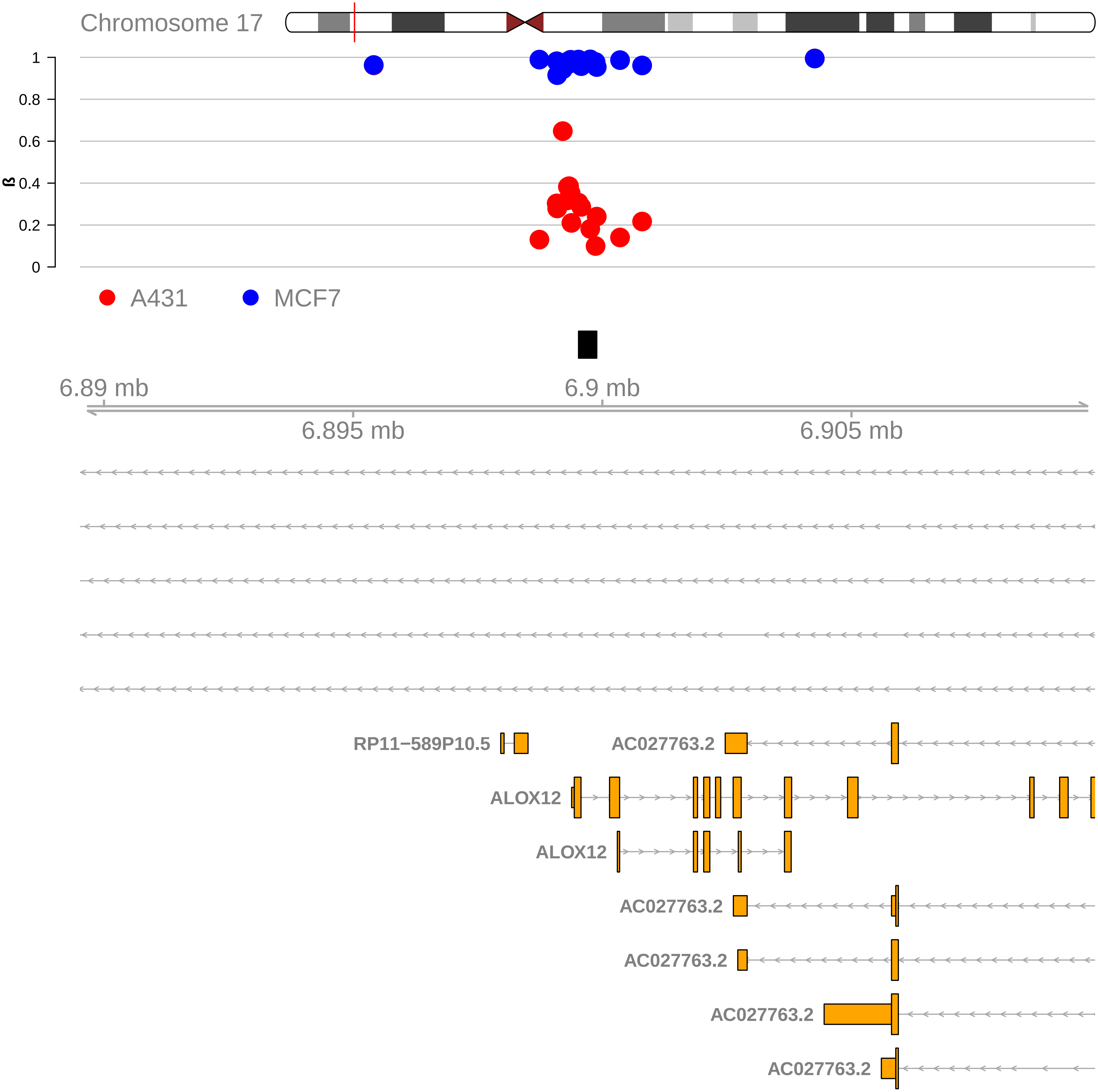

Chromosome 17
1
0.8
0.6
β 0.4
0.2
0
A431
MCF7
6.89 mb
6.9 mb
6.895 mb
6.905 mb
RP11−589P10.5
AC027763.2
ALOX12
ALOX12
AC027763.2
AC027763.2
AC027763.2
AC027763.2
